# Percutanous Electrochemotherapy (ECT) in Primary and Secondary Liver Malignancies: A Systematic Review

**DOI:** 10.3390/diagnostics13020209

**Published:** 2023-01-05

**Authors:** Vincenza Granata, Roberta Fusco, Valeria D’Alessio, Igino Simonetti, Francesca Grassi, Lucrezia Silvestro, Raffaele Palaia, Andrea Belli, Renato Patrone, Mauro Piccirillo, Francesco Izzo

**Affiliations:** 1 Division of Radiology, Istituto Nazionale Tumori IRCCS Fondazione Pascale—IRCCS di Napoli, 80131 Naples, Italy; 2 Oncology Medical and Research & Development Division, Casalnuovo di Napoli, 80013 Naples, Italy; 3 Division of Radiology, Università degli Studi della Campania Luigi Vanvitelli, 80127 Naples, Italy; 4 Division of Clinical Experimental Oncology Abdomen, Istituto Nazionale Tumori IRCCS Fondazione Pascale—IRCCS di Napoli, 80131 Naples, Italy; 5 Division of Epatobiliary Surgical Oncology, Istituto Nazionale Tumori IRCCS Fondazione Pascale—IRCCS di Napoli, 80131 Naples, Italy

**Keywords:** Electrochemotherapy, liver, percutaneous approach

## Abstract

The aim of the study was to analyse papers describing the use of Electrochemotherapy (ECT) in local treatment of primary and secondary liver tumours located at different sites and with different histologies. Other Local Ablative Therapies (LAT) are also discussed. Analyses of these papers demonstrate that ECT use is safe and effective in lesions of large size, independently of the histology of the treated lesions. ECT performed better than other thermal ablation techniques in lesions > 6 cm in size and can be safely used to treat lesions distant, close, or adjacent to vital structures. ECT spares vessel and bile ducts, is repeatable, and can be performed between chemotherapeutic cycles. ECT can fill the gap in local ablative therapies due to being lesions too large or localized in highly challenging anatomical sites.

## 1. Introduction

Liver cancer is the sixth leading cause of tumours worldwide, and the fourth for cancer deaths. The reason for the increase in the number of new cases of liver cancer is probably due to changing risk factors, such as obesity, alcohol consumption, and chronic infections with hepatitis B and C, diabetes type II, aflatoxin B1 [1,2]. The liver is also the most frequent site of metastasis from colon and rectum cancer [1] and the management of these malignancies is multidisciplinary. The surgical approach represents the gold standard for patients with primary or secondary liver cancer, but only 25% of these patients are eligible for surgery [3,4] due to factors such as age, pathological condition, severity of disease, location and size of lesions, insufficient proportion of the liver remaining after surgery, or the patient’s own decision [5,6,7,8,9,10,11,12,13,14]. In recent years, minimally invasive treatments for liver metastases have been developed (both thermal and not thermal) for liver metastases, and now, local treatment represents an option for the treatment of these patients.

Local treatment of metastatic colorectal cancer (CRC) has been included in consensus guidelines by the European Society of Medical Oncology (ESMO) [15]. Percutaneous ablation techniques are performed under imaging guidance using specific applicators of different diameter and shape [16,17,18,19]. The shape of the electrodes and the amount of energy delivered affect the ablation volume that depends also on the tumour environment and proximity to a large vessel that leads to the heat sink effect [20].

Thermal ablation techniques, including radiofrequency ablation (RFA), microwave ablation (MWA), and cryoablation (CRYO) are used for the treatment of primary and secondary liver tumours. The choice depends on lesion numbers, their localization, size, and local tumour environment. Both RFA and MWA cause a 3-fold elevation of temperature in the target tissue causing the coagulative necrosis of targeted cells directly or indirectly with desiccation of the tissue and destruction of microvasculature [21]. CRYO causes a drop in temperature below −40° within the target tissues and induces a freeze–thaw cycle due to the expansion of argon gas, leading to cell death in a small radius near the probe [21]. 

Each technique has its advantages and disadvantages and the choice of which to employ depends on the size of the tumour that must be at a maximum of 3.5 cm to be treated with RFA, or 5 cm with MWA and CRYO [21,22,23,24,25].

Non-thermal techniques include trans-arterial therapy (TAE) or trans-arterial che-moembolization (TACE). Both procedures are suitable for patients with unresectable disease involving less than 50% of the liver and without cirrhosis [26]. Tumours within the main portal vein, biliary obstruction and hepatic encephalopathy are contraindications for these treatments. These procedures are generally suggested to patients with palliative intent when curative treatment is not possible.

ECT is a not thermal local ablative procedure that by transient pore formation in the cellular membrane created by electroporation is able to induce enhancement of drug delivery in target cells [27,28]. ECT has been largely used, according to the Standard Operating Procedure developed by the European Standard Operating Procedure for Electrochemotherapy (ESOPE) with the Cliniporator ^TM^ Device (IGEA SpA, Carpi, Italy), to treat cutaneous and subcutaneous tumours, and mucosal tumours regardless of histology and body location [28,29,30,31].

The feasibility, safety and effectiveness of ECT has also been shown in deep-seated tumours, such as liver tumours [32,33,34,35] and metastases located near large liver vessels [36,37]. ECT is well-tolerated, with limited side effects [38,39,40]. ECT is specifically suitable for the treatment of liver metastases located centrally, close to the capsule or in proximity of the major vessels, which are not resectable and unsuitable for radiofrequency ablation or microwave ablation due to the heat sink effect [40,41,42,43,44,45,46]. 

The aim of our study was to analyse papers describing the use of ECT in the local treatment of primary and secondary liver tumours at different sites and with different histologies. In addition, ECT is compared with other thermal ablative percutaneous techniques.

## 2. Methods

This review is the result of a self-study without protocol and registration number. PRISMA guidelines were used for this systematic review. 

In order to ensure an adequate variety of the assessed studies, several electronic databases were considered: PubMed (US National Library of Medicine, http://www.ncbi.nlm.nih.gov/pubmed accessed on 15 October 2022), Scopus (Elsevier, http://www.scopus.com/ accessed on 15 October 2022), Web of Science (Thomson Reuters, http://apps.webofknowledge.com/ accessed on 15 October 2022) and Google Scholar (https://scholar.google.it/ accessed on 15 October 2022). 

Only clinical studies published between 2011 and 2022 were analysed, considering this time window consistent with the recent developments concerning the Electrochemotherapy application. Papers not indexed in the electronic databases were evaluated through the references of included studies. Eight [44,47,48,49,50,51,52,53] out of 16 studies were selected. Preclinical studies, review articles and case reports with a single enrolled patient were excluded. However, case reports could be reported in the Section 4.

**Figure 1:**
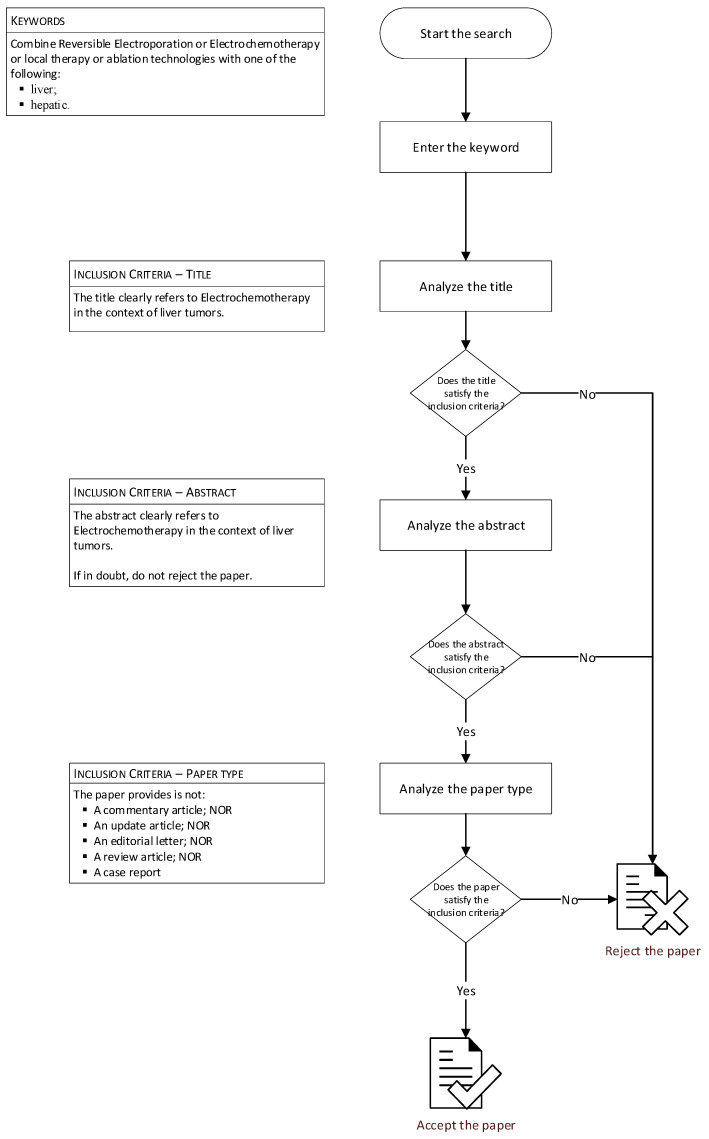
Flowchart of research methods.

The selection of papers was made by two reviewers according to a specific procedure (Figure 1). Only papers that met the inclusion criteria and which were written in English were considered. The two investigators extracted data from the included papers and recorded the number of patients treated with ECT, lesion size, percentage of lesions localized in challenging location, and local tumour control. Overall survival was added when available.

## 3. Results

Data collected from all the papers included in the analysis are summarized in Table 1. Table 2 shows the reported side effect for each manuscript. For each study, Electrochemotherapy was performed using the electric protocol defined by ESOPE guidelines (electric pulses of 100 µsec at 1000 V/cm) and Bleomycin was administrated intravenously (15,000 IU/m^2^). Linear fixed configuration, hexagonal fixed configuration or variable geometry manufactured by IGEA S.p.A. using multiple insertion of a single needle was used in the studies as reported in Table 1. Electric pulses were delivered using a generator (IGEA S.p.A., Italy) with the following parameters: 8–96 pulses at 400–3000 V (910–1000 V/cm), of 100 μs duration, at 1–5000 Hz of repetition frequency or a single pulse for a single relived R-wave (ECG synchronization). No combined treatments with ECT were reported by each included study.

The first evidence of feasibility, safety, and efficacy of intraoperative ECT in the treatment of colorectal liver metastases was published by Edhemovic et al. [44]. Twenty-nine metastases in 16 patients were treated in 16 sessions of ECT. Radiological evaluation of all the treated metastases showed 85% complete responses and 15% partial responses. In a group of seven patients that underwent a second operation at 6–12 weeks after the first one, during which ECT was performed, the histology of resected metastases treated by ECT showed less viable tissue (*p* = 0.001) compared to non-treated ones. No immediate (intraoperative) and/or postoperative serious adverse related events were observed. No differences were observed in treatment responses in the central versus peripheral location of the lesions.

ECT has been already used to treat hepatocellular carcinoma (HCC) [45,46] and to treat a prospective case series of patients with liver cirrhosis and Vp3-Vp4-portal vein tumour thrombus (PVTT) from hepatocellular carcinoma (HCC). In patients with cirrhosis, ECT seems effective and safe for curative treatment of Vp3-Vp4 PVTT from HCC [47]. All patients underwent three-phase computed tomography (CT), contrast enhanced ultrasound (CEUS) and ultrasound-guided percutaneous biopsy of the thrombus before ECT. CEUS examination after treatment showed a complete absence of enhancement of the treated thrombus in all cases. Post-treatment biopsy showed apoptosis and necrosis of tumour cells in all cases. Follow-up ranged from 9 to 20 months (median, 14 months). In two patients, the follow-up CT and CEUS showed complete patency of the treated portal vein without any intravascular or perivascular recurrence during follow-up. The other three patients had a persistent avascular non-tumoral shrinked thrombus at CEUS and CT during follow-up. No local recurrence was observed at follow-up CT and CEUS in 5/6 patients. In the remaining patient, 24 h post-treatment CEUS showed an absence of enhancement of the treated thrombus, but this patient was lost for follow-up because of death from gastrointestinal haemorrhage 5 weeks after ECT. The high risk of haemorrhage from gastroesophageal varices after ECT treatment of the main, right or left PV must be considered in the pre-treatment evaluation of the patients [47].

ECT was also used to treat perihilar cholangiocarcinoma (PHCCA) [48]. Five patients with PHCCA underwent ECT. Three patients underwent percutaneous ECT of a single PHCCA nodule. One patient underwent resection of a nodule in the IV segment and intraoperative ECT of a large PHCCA in the VIII segment. Another patient underwent percutaneous ECT of a large PHCCA recurrence after left lobectomy and RF ablation of a synchronous metastasis in the VI segment. The CT evaluation at 4 weeks post-treatment showed a complete response in three cases and incomplete response in two cases. The follow-up ranged from 10 to 30 months. Two of these five patients were alive at 30 months, with no local or distant intrahepatic recurrences in other segments. The case series was the first study to investigate the safety and efficacy of ECT in the treatment of patients with inoperable PHCCA. It demonstrates that ECT is feasible, safe, and effective and may be a suitable option for the treatment of PHCCA in selected clinical situations.

Electrochemotherapy with bleomycin was recently performed by Djokic et al. on 17 hepatocellular carcinomas in 10 patients [49]. The median size of the treated lesions was 24 mm (range 8 and 41 mm), located either centrally, that is, near the major hepatic vessels, or peripherally. The complete response rate at 3–6 months was 80% per patient and 88% per treated lesion. At the last observation (medium observation time of 20.5 months), the results showed a complete response in 15 out of 17 lesions. ECT is predominantly applicable in patients with impaired liver function due to liver cirrhosis and/or with lesions where a high-risk operation is needed to achieve curative intent, given the intra/perioperative risk for high morbidity and mortality [49]. ECT of hepatocellular carcinoma proved to be a feasible and safe treatment in all 10 patients included in this study.

A prospective pilot study to evaluate the feasibility, safety, and efficacy of intraoperative ECT for otherwise unresectable colorectal liver metastases was performed by Coletti et al. [50]. In this study, five patients with nine colorectal liver metastasis of dimension < 3 cm were treated with ECT with bleomycin pre-treatment followed by open liver resection [50].

Edhemovich et al. [51] demonstrated ECT’s long-term effectiveness and safety in a prospective study on 39 patients with unresectable metachronous colorectal liver metastases. In this paper, the authors reported an objective response rate equal to 75% (63% of complete response, 12% of partial response) and a median duration of the response equal to 20.8 months for metastases in a complete response and 9.8 months for metastases in a partial response. The therapy was significantly more effective for metastases smaller than 3 cm in diameter than for larger ones. There was no difference in response according to the metastatic location, that is, metastases in central vs. peripheral locations. 

However, there was no difference in overall survival for metastases smaller than 3 cm in diameter than for larger ones, with a median overall survival time of 29.0 months. 

Local tumor control was evaluated in patients with liver malignancies treated by local ablative therapies (LAT). Target lesions were characterised by histology, dimensions in three spatial axes, volume, vascularisation and challenging (CL) location. RFA, MWA, CRYO, ECT and Interstitial Brachytherapy (IBT) were used for local treatment. The study included 155 patients and 211 LAT were performed. Follow-up including MRI for all patients was 11 months. Larger lesions were treated with ECT and IBT and were significantly more often located in challenging location in comparison to those treated with RFA, MWA and CRYO. Best local tumor control (LTC) at 12 months resulted after RFA (93%), followed by ECT (81%), CRYO (70%), IBT (68%) and MWA (61%). Depending on the primary, best results were observed for hepatocellular carcinoma (HCC) (93%), followed by CRC (83%) and breast cancer (BrC) (72%), without statistically significant differences. Local tumor control at 12 months was higher for hypervascular lesions (92% *p* = 0.07) followed by intermediate (82% *p* = 0.01) and then hypovascular lesions (64%). Neither diameter nor challenging location had a significant impact on local tumor control even if a tendency to decrease was observed in the larger lesions. In challenging location, the best LTC resulted after RFA (82%) ECT (76%) and IBT (76%). [52]

Spalleck et al. [53] performed a retrospective analysis of patients with liver tumors or metastases treated with percutaneous ECT. Eighteen consecutive patients with measurable liver tumors of different histopathologic origins, mainly colorectal cancer, breast cancer, and hepatocellular cancer were recruited. Only mild or moderate side effects were observed after ECT. The objective response rate was 85.7% (complete response 61.9%, partial response 23.8%), the mean progression-free survival (PFS) was 9.0 ± 8.2 months, and the overall survival (OS) was 11.3 ± 8.6 months. In Table 1, the PFS and OS per different sizes of lesions are shown. ECT performed best (PFS and OS) in lesions within 3 and 6 cm diameters (*p* = 0.0242, *p* = 0.0297 respectively). PFS was higher for patients with lesions with lesions < 6 cm (12.0 ± 9.2 months) vs. patients with lesions > 6 cm (4.7 ± 5.4). The lesion localization distant, close or adjacent to vital structures did not influence the effectiveness of ECT. Progression-free survival and overall survival were independent of the primary histology considered. These results seem to demonstrate that ECT is an effective and valuable option for the treatment of unresectable liver metastases that cannot be treated with other ablative techniques.

Analysis of these studies demonstrate that ECT is effective in the treatment of liver lesions (metastases or primitive cancers) of different origin, size and localization, providing a long-term local tumor control method as well as long progression-free survival.

## 4. Discussion

A wide range of therapeutic options has been developed for treatment of primary and secondary liver tumors to compensate for the limited effectiveness of systemic therapies [54,55,56,57,58,59,60,61,62,63,64,65]. 

Surgical resection and/or transplantation, as well as local ablative therapies and regional or locoregional therapies, have been validated by prospective studies demonstrating improved patient survival. Surgical resection is the optimal therapeutic option when patients are suitable. However, surgical resection can lead to complications in the presence of cirrhosis or prolonged chemotherapy.

Liver loco-regional treatments, like trans-arterial chemoembolization (TACE) or radio embolization (TARE), have been employed for the treatment of unresectable intrahepatic metastasis (IM) with benefit on overall survival. For large or multinodular tumours locoregional therapies are preferred if the liver function is preserved [65,66,67,68,69,70,71,72,73,74,75,76,77,78,79,80]. Thermal ablation techniques including RFA, MWA and CRYO, are used for treatment of primary and secondary liver tumors. Local ablation has minimal morbidity, lower cost and shorter hospital stays [81]. Each local therapies have its advantages and disadvantages and the choice depends by size of the lesion and location with the aim to induce localized cytotoxicity of the tumor preserving near normal tissue [82,83,84,85,86,87,88,89,90,91,92].

Electrochemotherapy of colorectal liver metastases has proven to be a feasible, safe, and efficient treatment method. It allows the treatment of metastases located near the major hepatic vessels that cannot be removed by surgery or radiofrequency ablation. 

In addition, ECT treatment of deep-seated tumors of colorectal liver metastases does not affect cardiac function, and no major cardiac rhythm changes or pathological morphological changes were observed. Ten patients treated with ECT and monitored with Holter electrocardiographic (ECG) signals during the periods of 24 h before and after the surgical procedure involving ECT showed only minor significant but clinically irrelevant changes in heart rate and long-term heart rate variability (HRV) parameters during intra-abdominal ECT treatment [92]. 

ECT has already used to treat hepatocellular carcinoma [45,46] and was also safe and effective for treatment of patients with liver cirrhosis and Vp3-Vp4-portal vein tumor thrombus (PVTT) from hepatocellular carcinoma [47]. ECT represents a suitable option for the management of PHCCA in selected clinical settings, as shown by Tarantino et al. [48]. These results were confirmed in a 71-year-old male affected by a CCA at hepatic hilum and treated with ECT according to ESOPE guidelines. No complications occurred during the ECT procedure and no progression of the disease was found at 18 months with a computed tomography (CT) assessment [93]. 

In recent years, the combination of ECT for intrahepatic metastases (IM) and cytoreductive surgery (CRS) with hyperthermic intraperitoneal chemotherapy (HIPEC) has been used in a small number of cases with encouraging results. A first synchronous application of ECT and CRS and HIPEC to treat a patient with IM and intraperitoneal metastases (PM) from CCA was described by Stefano et al. [94] A man (47 yrs old) with CCA underwent hepatic resection and systemic therapy. After 14 months, for the occurrence of IM, the patient underwent a second hepatic resection and other chemotherapy cycle. Nonetheless, after 38 months from the first HR a new recurrence occurred and cytoreductive surgery and HIPEC with cisplatin and mitomycin for PM and ECT with BLM on a bulky metastasis of the hepatic hilum were performed. At the computed tomography performed 11 days after treatment complete necrosis of the treated IM was detected. CT scans after 3 and 6 months and magnetic resonance after 9 months showed necrosis of the treated IM and PM, but progression of the residual liver lesions was observed. After 3 months, the patient received SC and underwent TACE after 8 months and TARE after 9 months for the residual liver metastases. At 14 months from CRS and HIPEC, the patient was alive, in good condition, and with stability of the disease [94].

We conducted our study with the intention of determining whether the use of ECT may be beneficial compared with other local thermal techniques in the treatment of particularly large lesions and lesions located in particularly difficult sites.

Combination of local ablative therapy and other options have been explored such as RFA and TACE [95]. Several meta-analyses accumulated data from randomized clinical trials available for RFA plus TACE suggesting that the combination of TACE with RFA improved outcomes compared to RFA alone [96,97,98]. 

Our attention has mainly focused on liver metastases originating from colorectal cancer, but also metastases of different origin were included in our selection [99,100,101]. We analysed height studies in which ECT or other thermal local procedures were used to treat primary and secondary liver tumors. In these studies, larger lesions were predominantly treated with ECT or IBT while smaller lesions were treated with RFA, CRYO and MWA. Lesions located in challenging positions were treated using ECT rather than with other local ablative therapies. The best local tumor control method depending on the primary tumor was obtained with HCC followed by CRC and BrC, but no statistically significant differences were highlighted.

Local tumor control seems to be higher in hypervascular lesions in comparison to hypovascular ones and not dependent on diameter or volume nor on challenging locations of the target lesion [52], thanks to the careful pre-selection of the most suitable therapy based on current evidence. 

Then, large lesions are not an obstacle to local ablative therapies if the right technique and probe are chosen. In the vicinity of large vessels, a method such as ECT, which is not limited by the heat sink effect, can be used safely.

A limitation of this study is the small number of papers. Because of the limited number of studies, it was not possible to perform a subgroup analysis for primitive and secondary liver tumors. However, the eight articles identified are the only ones that met the included criteria. They may be useful and sufficient to provide readers with systematic preliminary results on percutaneous electrochemotherapy for primary and secondary liver malignancies.

## 5. Conclusions

Analysis of this work shows that ECT is safe and effective in large lesions, regardless of the histology of the lesions treated. ECT performed better than other thermal ablation techniques in lesions > 6 cm and can be safely used to treat lesions that are near or adjacent to vital structures. ECT spares vessels and bile ducts, is repeatable, and can be performed between chemotherapy cycles. ECT can fill the gap in local ablative therapy for lesions that are too large or for lesions in very difficult anatomic locations.

## Figures and Tables

**Table 1:** Summary table of the studies analysed in this manuscript.

Paper	N. of Patients (Lesions)	Number of Treatment with Fixed or Variable Geometry	Dimension of Lesions	Type of Cancer (% of Cases)	Challenging Location	Local Tumour Response		Overall Survival (Months) Mean ± Standard Deviation
Edhemovich et al. [44]	16 (29) secondary liver tumors	Fixed (n. 6) and variable geometry (n. 10)	<3.0 cm	CRC liver metastases	48% of lesions	CR 85% PR 15%		Not available
Tarantino et al. [47]	6 primitive liver tumors	Variable geometry (n. 6)	2.5–4.5 cm	HCC	100% of lesions	CR 100%		16.6% at 20 months
Tarantino et al. [48]	5 primitive liver tumors	Variable geometry (n. 5)	3.0–6.0 cm, mean = 4.2 cm	Cholangio-carcinoma	100% of lesions	CR 60%		40% at 30 months
Diokic et al. [49]	10 primitive liver tumors	Fixed (n. 5) and variable (n. 3) and both (n. 2) geometry	0.8–4.1 cm	HCC	100% of lesions	CR 88%		Not available
Coletti et al. [50]	5 (9) secondary liver tumors	Fixed geometry (n. 9)	mean 2.6 cm (range 0.6–3.0 cm)	CRC liver metastases	Not available	CR 55% SD 45.5%		100% at 6 months
Edhemovich et al. [51]	39 secondary liver tumors	Fixed (28) and Variable (11) geometry	mean 2.0 cm (range 0.3–6.0 cm)	CRC liver metastases	Not available	CR 63% PR 12% SD 2% PD 23%		29 months (median)
Kovacs et al. [52]	8 primitive liver tumors and 13 secondary liver tumors	Variable geometry (n. 21)	<3 cm 5% 3.0–6.0 cm 38% >6.0 cm 57% <10 cm 5%	HCC (14%), CRC liver metastases (38%), BrC (24%): other primary tumour (24%);	91% of lesions	ORR: HCC 93% CRC 83% BrCa 72%		OS at 12 months: HCC 83% CRC 62% BrCa 64%
Spalleck et al. [53]	2 primitive liver tumors and 16 secondary liver tumors	Variable geometry (n. 18)	3.0–6.0 cm	CRC (39%), BrCa (22%), HCC (11%), Ovarian (11%), Anal (0.5%), NSCLC(0.5%), unknown origin (0.5%)	90.5% of lesions	All lesions	For lesion < 6 cm CR 90% PR 0% SD 0% PD 0% For lesion > 6 cm CR 36.4% PR 45.4% SD 9.1% PD 0%	For lesion < 6 cm Overall survival 15.1 ± 8.0 months For lesion < 6 cm Overall survival 7.9 ± 7.9 months
						CRC lesions	CR 50%, PR 25%, SD 0% PD 0%	
						BrCa lesions	CR 80%, PR 20%, SD 0%, PD 0%	
						HCC lesions	CR 33.3%, PR 66.7%, SD % 0, PD 0%	

Note: CR: Complete Response; PR: Partial Response; SD: Stable Disease; PD: Progression Disease; ORR: Overall Response Rate; HCC: Hepatocellular carcinoma; CRC: Colorectal carcinoma; BrCa: Breast Carcinoma; NSCLC: Non-Small Cell Lung Carcinoma.

**Table 2:** Reported side effect for each manuscript.

Paper	Reported Side Effect
Edhemovich et al. [44]	Fever: Two patients
Tarantino et al. [47]	No intraoperative or post-operative major complication
Tarantino et al. [48]	None
Diokic et al. [49]	No intraoperative or postoperative complications during the first 24 h occurred. Two patients presented transient ascites
Coletti et al. [50]	Wound dehiscence: One patient Bowel occlusion: One patient eight days after surgery
Edhemovich et al. [51]	None
Kovacs et al. [52]	None
Spalleck et al. [53]	Mild pain: 16 patients Protein C elevation and leucocytosis: One patient Liver capsular hematoma: One patient

## Data Availability

Data are available in the manuscript and at link https://zenodo.org/record/7503110#.Y7U_y3bMK3A, accessed on 30 December 2022.

## Abbreviations

LAT	Local Ablative Therapies
ECT	Electrochemotherapy
CRC	Colorectal Cancer
ESMO	European Society of Medical Oncology
RFA	Radiofrequency ablation
MWA	Microwave ablation
CRYO	Cryoablation
TAE	Transarterial therapies
TACE	Transarterial chemoembolization
ESOPE	European Standard Operating Procedure for Electrochemotherapy
CR	Complete Response
PR	Partial Response
PD	Progression Disease
SD	Stable Disease
ORR	Overall response rate
HCC	Hepatocellular carcinoma
BRCA	Breast Carcinoma
OS	Overall survival
CT	Computed tomography
CEIS	Contrast enhanced ultrasound
PHCCA	Perihilar cholangiocarcinoma
LTC	Local tumour control
NSCLC	Non-Small Cell Lung Carcinoma
TARE	Radioembolization
PFS	Progression free survival
HIPEC	Hyperthermic Intraperitoneal chemotherapy
IM	Intrahepatic metastases
PM	Intraperitoneal metastases
